# Effectiveness of oral health promotion program for persons with severe mental illness: a cluster randomized controlled study

**DOI:** 10.1186/s12903-020-01280-7

**Published:** 2020-10-27

**Authors:** Mei-Wen Kuo, Shu-Hui Yeh, Heng-Ming Chang, Po-Ren Teng

**Affiliations:** 1 Department of Psychiatry, Chang Bing Show Chwan Memorial Hospital, No. 6, Lugong Rd., Lugang Township, Changhua County, 505 Taiwan, ROC; 2 Department of Nursing, Chang Bing Show Chwan Memorial Hospital, Changhua County, Taiwan, ROC; 3 Institute of Long-Term Care, MacKay Medical College, New Taipei City, Taiwan, ROC; 4 Orthodontic and Dental Department, Chang Bing Show Chwan Memorial Hospital, Changhua County, Taiwan, ROC

**Keywords:** Oral health, Promotion program, Plaque index, Mental illness

## Abstract

**Objective:**

To evaluate the effectiveness of a composite oral health promotion program designed to reduce dental plaque among persons with severe mental illness in a psychiatric institution.

**Methods:**

A cluster randomized controlled study was carried out in chronic psychiatric wards of a general hospital in central Taiwan. Sixty-eight eligible male individuals admitted to 2 wards were randomly assigned to an experimental and a control group. Participants in the experimental group underwent an oral health promotion program that consisted of biweekly group education sessions, and a 12-week individual behavioral modification for oral hygiene course. The participants in the control group received usual care only. Dental plaque (measured by the Plaque Control Index) was examined by a single dentist before and after the experiment. Each participant responded to a questionnaire regarding oral health knowledge, attitude and behavior before and after the experiment.

**Results:**

Fifty-eight individuals completed the study. Before the experiment, the plaque index was similar between the intervention group (68.9; N = 27) and the control group (69.8; N = 31). After the experiment, the plaque index was significantly better in the intervention group than in the control group (42.6 vs. 61.8; *P* < 0.001). Participants in the intervention group also demonstrated better oral health knowledge, attitude and behavior than those in the control group after the experiment.

**Conclusions:**

A composite oral health promotion program using both group education and individual behavioral methods over a 12-week period was effective in both reducing dental plaque and improving the oral health knowledge of persons with severe mental illness in the institution.

*Trial registration*: This study was retrospectively registered in Clinicaltrials.gov, with number NCT04464941, dated 7/7/2020. https://register.clinicaltrials.gov/RD103035018.

## Background

Severe mental illness (SMI) refers to psychiatric diseases that are so debilitating that a person's ability to engage in functional and occupational activities is severely impaired. Examples of common SMI are schizophrenia, bipolar disorder, major depressive disorder and organic mental illness. It is well documented that persons with SMI have higher rates of physical illness, including diabetes, cardiovascular disease, chronic lung disease, and cancer [1, 2]. This, in turn, increases mortality, with the life expectancy of schizophrenia patients reduced by up to 15–20 years over the general population. The greater prevalence of physical conditions [3, 4] among persons with SMI is most likely the result of lifestyle factors [5], such as smoking [6], eating a high-fat, low-fiber diet and exercising less [1], compared to the general population. To deal with this issue, a number of studies have been conducted with the aim of developing effective healthy living interventions in the areas of smoking cessation [7], weight management [8, 9], exercise [10] and nutritional education [11]. However, SMI patient compliance with long-term lifestyle interventions are often affected by negative symptoms, chaotic lifestyles and patient hospitalizations [12].

Compared to other physical diseases, there has been little attention paid to the oral health of patients with SMI. Studies have indicated that persons with SMI have a greater risk of developing oral disease [13, 14], which is linked to systemic diseases such as coronary heart disease, diabetes, hyperlipidemia, and respiratory disease [15–17]. Oral health can also affect the patient’s quality of life, self-esteem, speech, nutrition, and other social and psychological areas of life [18]. In Taiwan, studies show that the oral health of psychiatric inpatients is poor compared with the general population [13, 19]. Based on these cross-sectional surveys, the considerable unmet dental treatment need is observed from the high prevalence of dental caries, missing teeth and periodontal disease in patients with SMI [13, 20]. Nevertheless, oral health is often neglected by psychiatric personnel and the patients themselves [13]. Despite their worsening oral health, these patients receive less dental treatment than the general population [21, 22]. To date, only a few studies have investigated the effects of oral health education or interventions on people with SMI.

In one study, participants were randomly assigned to receive education, a reminder system and a mechanical toothbrush, or just a mechanical toothbrush, and the results indicated that the plaque index improved for both groups, but those that were given enhanced intervention showed much more improvement [23]. In another study, researchers compared the utility of motivational interviews with oral health education alone. The findings suggested motivational interviews were more effective than oral health education alone in enhancing short-term oral health behavioral change and oral hygiene status [24]. In both studies, there were no usual care groups, so the effects of interventions such as a reminding system or motivational interviews might be underestimated. In Korea, a dental hygiene care program using flash-based video, brochures and a toothpick method designed to reduce dental plaque in persons with mental disorders in a daytime mental health center found that the dental plaque index improved significantly in all subgroups, but the difference of effect size in different interventions were minimal [25].

Khokhar et al. conducted a systematic review to explore the effects of oral health education on people with serious mental illness. They concluded that there was no evidence that oral health advice helped those with SMI in terms of clinically meaningful outcomes [26]. Furthermore, they suggested that more good-quality studies are needed to obtain concrete evidence to aid in decision-making about the effectiveness of oral health interventions for those with SMI. Another study recommended that there be further research on the effects of more comprehensive oral health promotion programs with a non-intervention group [23]. To address this issue, we therefore designed a cluster randomized controlled trial to determine the effects of a composite oral health promotion program, including individual and group components, on patients with SMI.

The aims of this study were to evaluate the effects of an oral health promotion program on dental plaque and on any changes in patients’ oral health knowledge, attitude and behavior. We hypothesized that, first, patients in the intervention group would achieve more dental plaque reduction and, second, those who received education would enjoy improvements in their oral health knowledge, attitude and behavior.

## Methods

### Settings and subjects

This study was conducted in the psychiatric wards of a general hospital in central Taiwan. The hospital provides 4 psychiatric wards to serve 300 chronic psychiatric patients (75 patients in each ward). Two wards received only male patients who were randomly referred from acute wards after their acute symptoms subsided; therefore, the patient characteristics of the 2 wards were generally comparable. We used a cluster sampling method by computer sequence randomization to assign the patients in one chronic ward to be an intervention group and those in the other ward, a usual care group. This randomized controlled trial was performed in accordance with the CONSORT (Consolidated Standards of Reporting Trials) checklist (Additional file 1). Sample size was determined by power analysis. Variance estimates for the primary outcome measurement, plaque index scores, were based on the work of Almonani et al. [22], with a meaningful difference of 24% as effect size, and with a type I error at 0.05 and power at 0.80. The result showed that 30 subjects were needed to be recruited in each group with assumed participant dropout rate of approximately 30%. Individuals older than 65 years or younger than 20, and with significant cognitive disabilities, severe hearing impairment, orthodontic appliances, an edentulous status or a handicap when tooth-brushing were excluded. This study was approved by the Institutional Review Board of Show Chwan Memorial Hospital.

### Study procedure

This was a 12-week experimental study. Eligible subjects that provided informed consent were included in the study. Before the study, nurses in the ward selected as the intervention group received training in an accurate Bass tooth-brushing technique from the dentist, and in basic behavioral modification methods from a clinical psychologist. The subjects in the control group received no intervention, but were given usual nursing care, as before. The measurement of the dental plaque index was performed by one dentist who was blinded to the study assignment before and after the experiment. The dentist repeated the test on 10% of the tested subjects, and results of the dental plaque index rating indicated good intra-rater reliability.

### Intervention

The oral health promotion program was a composite intervention with both group and individual components. The group intervention consisted of:

1. Group oral health education: There were 5 sessions of group education including: structure of the oral cavity and teeth; importance of oral health; pathogenesis of caries and periodontal diseases; Bass tooth-brushing method; and proper oral hygiene. The group education session was conducted by trained nurses for 60 min in each session. The education sessions took place at interval of 2 weeks.
2. Display of Bass tooth-brushing methods: Pictures of the Bass tooth-brushing technique procedure were posted on the mirror in each bathroom of the participants’ rooms.
3. Broadcasting of songs as tooth-brushing reminders: We selected songs with a message of the benefits of tooth-brushing, and broadcasted them 5 times each day (upon awaking up, after each meal, and before going to sleep) to remind participants to brush their teeth.

The individual interventions included:

1. Instruction in the Bass tooth-brushing method: After each group education session, the participants received one-on-one training in the Bass tooth-brushing technique by trained nurses. All participants were checked for correctness of their brushing technique.
2. Individual behavioral modification method: Tokens were used to reinforce the act of tooth-brushing and adherence to participation in group meetings. After the nurses had checked for the accomplishment of successful tooth-brushing, the participants received a one-point token. In addition, participants were given 5-point tokens for their attendance at each group education session. The collected tokens could be used as money to purchase their daily goods (such as shampoo, soap or instant noodles).

### Measurement

#### Dental Plaque Index

All measurements were obtained at baseline and at 12 weeks following the intervention. We used the Plaque Control Record (PCR) developed by O’Leary et al. [27] to score the primary outcome, plaque accumulation, on the surfaces of the teeth. The score was computed by the existence of plaque divided by all examined surfaces; a higher plaque index indicated poor oral hygiene. We calculated a person-level plaque score by averaging plaque scores for all teeth at each examination. A single examiner, who was blinded to the group assignment, conducted all oral examinations.

#### Oral health knowledge, attitude and behavior

The original questionnaire was adapted from the tool proposed by the Taiwan Health Promotion School sponsored by the Ministry of Education in Taiwan. Then, a 35-item questionnaire related to oral health knowledge, attitude and behavior was developed and evaluated for face validity by a panel of 5 clinicians, and attained good content validity (content validity index greater than 0.8). A pilot-test with a sample of 30 patients with SMI was done to ensure that participants could understand the questions. Finally, there were 10 items with questions related to oral knowledge, 13 items related to oral attitude and 10 items related to oral behavior (Additional file 2). The scores of questions related to oral health knowledge ranged from 0 to 10, for those related to oral health attitude, 13–65, and for those related to oral health behavior, 0–10. The questionnaire possessed good internal consistency (Cronbach’s alpha coefficient = 0.87).

### Statistical analyses

Data were processed using SPSS version 18. The Chi-square test was used to compare proportions of 2 groups of categorical variables. The independent *t* test was used to compare the results of the intervention and control groups, in terms of dental plaque and questionnaire scores. The paired-*t* test was used to compare within groups difference after the experiment. Significance was set at 0.05.

## Results

**Fig. 1:**
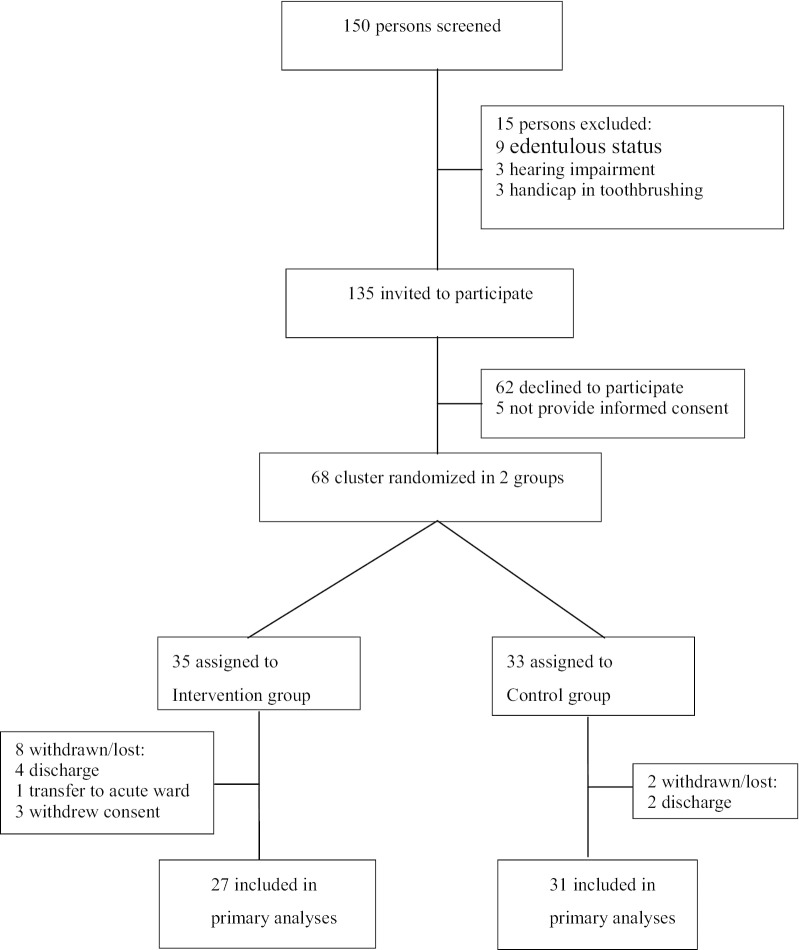
Participants flow chart

The participant flow chart can be seen in Fig. 1. Thirty-five patients participated in the experimental group and 33 in the control group. A total of 10 participants were discontinued from the study. There were no statistical differences in basic demographics between completers and drop-outs. The analysis was based on the completers, which included 27 participants in the experimental group and 31 in the control group. No adverse events were reported.

The majority of patients were middle-aged and had a high school education. Most patients suffered from some degree of xerostomia symptoms. Most of them consumed snacks every day and smoked cigarettes. The main mental illness diagnosis was schizophrenia. Baseline group comparisons revealed no significant group differences in terms of demographics (Table 1).

**Table 1:** General characteristics of participants

Characteristics	Intervention group (n = 27)		Usual care group (n = 31)		Total (n = 58)	
	n	%	n	%	n	%
Age group (year)						
20–29	1	3.7	4	12.9	5	8.6
30–39	11	40.7	13	41.9	24	41.4
40–49	10	37.0	10	32.3	20	34.5
50–59	4	14.8	3	9.7	7	12.1
≧60	1	3.7	1	3.2	2	3.4
Education (year)						
Elementary school	2	7.4	1	3.2	3	5.2
Junior high school	8	29.6	13	41.9	21	36.2
Senior high school	10	37.0	13	41.9	23	39.7
College +	7	25.4	4	12.9	15	19
Oral dryness						
No	8	29.6	10	32.3	18	31.0
Occasional	16	59.3	16	51.6	32	55.2
Frequent	3	11.1	5	16.1	8	13.8
Snack habit						
No	3	11.1	3	9.7	6	10.3
Once per day	9	33.3	13	41.9	22	37.9
Twice per day	10	37.0	11	35.5	21	36.2
Twice per day	4	14.3	1	3.2	5	8.6
Fourth or more	1	3.7	3	9.7	4	6.9
Smoking habit						
Yes	12	44.4	16	51.6	28	48.3
No	11	40.7	11	35.5	22	37.9
Quit	4	14.3	4	12.9	8	13.8
Mental illness						
Schizophrenia	24	88.9	27	87.1	51	87.9
Mood disorder	1	3.7	0	0	1	1.7
Organic mental illness	0	0	4	12.9	4	6.9
Others	2	7.4	0	0	2	3.4

No significant difference in each characteristic by Chi-Square test

### Dental Plaque index

**Fig. 2:**
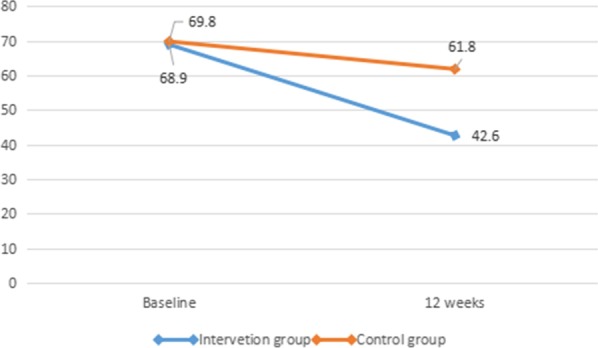
Changes in dental plaque index by group

Before the study, the mean dental plaque index in the intervention group was 68.9, and in the control group, 69.8, with no significant difference (*P* = 0.776). After the 12-week intervention effort, the mean dental plaque index of the intervention group (42.6) (standard deviation, SD = 12.1) was significantly improved, compared to that of the control group (61.8) (SD = 11.6) (*P* < 0.001) (Fig. 2). The dental plaque index also improved significantly in both the control group (mean difference: 8.0; *P* < 0.001) and the intervention group (mean difference: 26.3; *P* < 0.001).

### Oral health knowledge, attitude and behavior

Before the intervention, the participants had similar results in terms of oral health knowledge, attitude and behavior. Among subjects in the intervention group, the oral health knowledge score improved from 4.6 to 8.5, the oral health attitude score improved from 47.4 to 58.6, and the oral health behavior score improved from 4.1 to 8.1. In all 3 domains, participants in the intervention group demonstrated statistical improvement, compared to those in the control group (*P* < 0.001) (Table 2). Based on the questionnaire on oral health behavior, although the intervention group showed a significant improvement in tooth-brushing behavior, there was no significant difference between the intervention group and control group in terms of sugary beverage consumption habit and dentist-visiting behavior.

**Table 2:** Change of oral health knowledge, attitude and behavior

	Oral health knowledge	*P* value	Oral health attitude	*P* value	Oral health behavior	*P* value
Before study						
Intervention group	4.6	0.54	47.4	0.38	4.1	0.45
Control group	4.3		45.9		3.6	
After study						
Intervention group	8.5	< 0.001	58.6	< 0.001	8.1	< 0.001
Control group	4.7		45.8		3.8	

Range of score of oral health knowledge 0–10; oral health attitude 13–65; oral health behavior 0–10

## Discussion

The results of this study provide initial evidence of the benefits of an oral health promotion program for improving the oral hygiene status of patients with SMI. Moreover, patients with SMI may enjoy improvement in oral health knowledge, attitude and behavior after just a short period of group education. To the best of our knowledge, this is the first report using both group and individual components of an oral health intervention to achieve oral hygiene improvement in patients with SMI. The strengths of the study included its randomized controlled study design, that the examiner was blinded to group assignments, and there was an adequate sample size and a low drop-out rate.

To cope with the universally poor oral health conditions among patients with SMI, multiple levels of strategies have been proposed, consisting of reorganization of dental services [28], increased accessibility to dental care [13], regular dental check-ups [21, 29], undertaking aggressive preventive education programs [30], and improving oral health hygiene by means of educational intervention [31]. However, psychiatric patients are known to suffer from a variety of so-called negative symptoms, such as apathy and loss of drive, which would impede self-care and lead to neglect of oral hygiene [13]. Some cognitive-behavioral approaches have been tried. In 2 studies by Almomani et al., oral health education, a reminder system, provision of a mechanical toothbrush [23] and motivational interviewing [24] were used. In a study by Mun et al., an oral healthcare education program using video and brochures was provided to participants [25]. In the current study, however, we used a combination of cognitive-behavioral methods and a group format.

With regard to experiences in applying general lifestyle interventions to persons with SMI, the literature has indicated that single-component programs are less effective than those employing multiple components [32, 33]. Moreover, group sessions are potentially cost-effective and may be beneficial in reducing social isolation in this population [34]. Mental illness can compromise a person’s ability to make decisions in daily life, and so social support and peer support are essential to learning about and practicing oral health programs tailored for those patients [35]. In chronic psychiatric wards, as with our study setting, education, reminder systems, and behavioral modification measures are employed on a group basis, and we postulated that the group effect could have a motivational effect on psychiatric inpatients to encourage them to adhere to oral health programs and ultimately improve their oral hygiene.

In Japan, Yoshii et al. conducted a study to determine the effect of an educational program on improving oral health self-care. The one-time 30-min oral hygiene education presentation resulted in an improvement in the use of fluoride toothpaste and in the daily use of interdental brushes or floss, even 6 months after the intervention [31]. However, the habit of tooth-brushing or visiting the dentist remained unchanged after the educational program. In our study, the group education program was applied repeatedly, 5 times in all, and was combined with a behavioral change strategy. The results of the Yoshii et al. study indicated that education alone might not bring sustained change to the oral hygiene habits of patients with SMI, and that a combination of both cognitive and behavioral therapeutic interventions are needed for persons with SMI. Furthermore, to achieve sustained oral health hygiene behavioral change, repeated educational sessions should be provided, instead of only a few interventions.

Strategies to improve the oral health of persons with SMI in the community would be different from those applied in psychiatric wards. Preventive and treatment programs for oral health should be tailored to meet the individual needs of patients based on their diagnoses, severity of mental illness and cognitive functions [36]. It has been suggested that cognitive function may play a crucial role in dentist-seeking behavior among patients with SMI [21]. Oral hygiene habits, easily neglected by patients with SMI, should be promoted by not just education, but more proper measures, such as behavioral modification, to deal with the negative symptoms and poor cognitive functions of those patients.

In the current study, individual behavioral modification was encouraged by token reinforcement contingent on tooth-brushing behavior or attendance at group sessions. In the literature on healthy living interventions, monetary payment or tokens were given to psychiatric patients who abstained from smoking [37, 38] or lost weight [9]. Our results are consistent with previous studies that demonstrated the effectiveness of contingent management approaches in reinforcing healthy behavioral change in persons with SMI. However, the generalizability of contingency behavioral approaches might not be replicable in routine health care settings, since there is a high degree of control of the environment in institutions. Other individual-level approaches such as the use of praise and disapproval contingent on targeted behavioral change might be considered unethical and possibly iatrogenic [39].

Our study result is consistent with that of Almomani et al., in that persons with mental illness had greater improvement in oral health knowledge after the education sessions [24]. Previous studies suggest that poor oral hygiene may reflect a lack of knowledge or the lack of a rationale for treatment [40, 41]. Therefore, knowledge acquisition through education may be an important step to take in changing the oral self-care behavior of those patients. In our study, only an instructive educational program was used with the participants. Peteuil et al. proposed a therapeutic educational program that used focus group interactions to achieve better oral health knowledge and oral health-related quality of life [42]. To date, there is no consensus on which educational intervention is better for persons with SMI. Further studies are needed to determine the most effective and convenient type of oral health education for these patients.

Most participants in the intervention group changed their oral health behavior, especially in terms of tooth-brushing. However, there was no significant difference in the consumption of sugar-added beverages and in dentist-visiting in both groups. A patient preference for snacks or sweet beverages might reflect their worse general health habits that are not easily changed by a few educational sessions. Additional efforts through motivational interviewing or environmental change (such as in supplying non-sweetened beverages or healthy snacks) should be taken to achieve better oral hygiene in patients with mental illness.

This was a short-term experimental study in which we observed improvements in dental plaque in those patients receiving the intervention only. Dental plaque, however, is only a surrogate end-point for our concern regarding this disadvantaged population, which is reducing the prevalence of caries and periodontal diseases. Nevertheless, there is no doubt that this is the first step we should take. Persons with SMI are susceptible to dental diseases for many reasons, including poor oral hygiene habits, reduced motivation for personal care, high consumption of sugary food or beverages, fear of dental treatment, neglect of oral health, difficulty accessing dental care facilities and reduced protective saliva due to the side effect of psychiatric medications [13, 14]. By improving the oral hygiene of patients with SMI, encouraging regular dental check-ups, increasing the accessibility of dental care, and increasing an awareness of the importance of oral health in patients and staff, as well, we can achieve better overall oral health and subsequently improve the general health of psychiatric patients.

The results of the present study should be interpreted with caution, in light of some limitations. First, the study subjects were psychiatric inpatients; therefore, the intervention measures taken to improve the oral health of persons with SMI might not be applicable to patients in the community. Further studies with a focus on designing appropriate interventions to achieve better oral health among patients in a variety of settings and with heterogeneous group characteristics should be conducted. Second, we noticed that dental plaque also improved among individuals in the control group. Given that the participants were not blinded to the group assignments, we cannot rule out the possibility of an extra-effort effect arising in the control group. But even so, the effect size of the intervention is still significantly large enough to substantiate the validity of the results. Third, the study intervention consisted of a composite of the elements of behavioral therapy, education and a group approach, so we are not certain which one was the key factor in reducing dental plaque. Finally, we did not measure the sustained effect of the oral health promotion program after the trial was finished. It is likely that further booster sessions are needed to help patients with SMI maintain good oral hygiene habits in the long term.

## Conclusion

In summary, an oral health promotion program using both group education and individual behavioral methods over a 12-week period was effective in reducing dental plaque and also in improving the oral health knowledge of persons with SMI in the institution. Further studies replicating the effectiveness of such approaches in settings outside the institutions are needed. In addition, a testing of the hypothesis that booster sessions are required ensure the sustained effect of such intervention is also needed.

## Supplementary information

**Additional file 1**. CONSORT checklist.

**Additional file 2**. Questionnaire of oral health knowledge, attitude and behavior.

## Data Availability

The dataset used and/or analysed during the current study are available from the corresponding author on reasonable request.

## Abbreviations

SMI: Severe mental illness
PCI: Plaque Control Index
