# Supplementary material for: Effectiveness of oral health promotion program for persons with severe mental illness: a cluster randomized controlled study
Source: BMC Oral Health. 2020 Oct 27;20:290. doi: 10.1186/s12903-020-01280-7 (PMC7590455; doi:10.1186/s12903-020-01280-7)
Supplement: Supplementary file 2 — Additional file 2. Questionnaire of oral health knowledge, attitude and behavior. [file 12903_2020_1280_MOESM2_ESM.doc]

| Topic 1. Knowledge of oral hygiene and health. Based on your understanding, please check in the box "□" the most appropriate answer to the following questions. | | |
| --- | --- | --- |
|  | True | False |
| 1. The main cause of tooth decay is bacteria in the teeth. | □ | □ |
| 2. When tooth decay has eroded the tooth enamel, eating cold or hot food can be painful. | □ | □ |
| 3. Periodontal diseases have nothing to do with smoking. | □ | □ |
| 4. Candies and cookies are better than vegetables and fruits at protecting teeth. |  |  |
| 5. Dental plaque is bacteria and their by-products attaching to the tooth surfaces. | □ | □ |
| 6. If I don't eat, it is not necessary to brush my teeth. | □ | □ |
| 7. Tartar is a stony growth on the teeth. | □ | □ |
| 8. The hardest outer layer of the teeth is dentin. | □ | □ |
| 9. The incisors are like knives for cutting meat and tearing food. | □ | □ |
| 10. The 333 Golden Rule of Brushing: Brush after each meal, brush within 30 minutes after a meal, and brush for at least three minutes each time. | □ | □ |

| Topic 2. Attitude toward oral hygiene and health. Based on your view toward oral health and your personal feelings, please check in the most appropriate box "□". | | | | | |
| --- | --- | --- | --- | --- | --- |
|  | Strongly Disagree | Disagree | Neutral | Agree | Strongly Agree |
| 1. I think that dental diseases can cause diseases in other parts of the body. | □ | □ | □ | □ | □ |
| 2. I think it is very important to brush my teeth immediately after a meal. | □ | □ | □ | □ | □ |
| 3. I don’t think it is okay to go to bed without brushing my teeth. | □ | □ | □ | □ | □ |
| 4. I think just brushing my teeth is enough to protect them, and brushing carefully or correctly is not that important. | □ | □ | □ | □ | □ |
| 5. I think if I rinse my mouth, I don't have to brush my teeth. | □ | □ | □ | □ | □ |
| 6. I think that as long as the bristles of my toothbrush are not bent or damaged, I can continue using it. | □ | □ | □ | □ | □ |
| 7. I don't go to the dentist because I am afraid of dental treatment. | □ | □ | □ | □ | □ |
| 8. I don't think I need to see a dentist if I don't have toothache. | □ | □ | □ | □ | □ |
| 9. I think that as long as I brush my teeth carefully, I don't have to floss them. | □ | □ | □ | □ | □ |
| 10. I think that if my cavity is not hurting, I don't have to do anything about it | □ | □ | □ | □ | □ |
| 11. The first step in dealing with gum bleeding is to clean my teeth well. | □ | □ | □ | □ | □ |
| 12. I think it is very important to have regular dental checkups. | □ | □ | □ | □ | □ |
| 13. I think brushing after every meal is a hassle. | □ | □ | □ | □ | □ |

| Topic 3. Oral hygiene and health behavior. In the following questions, check the box "□" according to your actual actions. | | |
| --- | --- | --- |
|  | Yes | No |
| 1. In the past week, did you brush your teeth for more than three minutes each time? | □ | □ |
| 2. In the past week, did you brush your teeth after getting up from bed? | □ | □ |
| 3. In the past week, did you brush your teeth after breakfast? | □ | □ |
| 4. In the past week, did you brush your teeth after lunch? | □ | □ |
| 5. In the past week, did you brush your teeth after dinner? | □ | □ |
| 6. In the past week, did you brush your teeth before going to bed? | □ | □ |
| 7. In the past week, did you clean your teeth (brush or floss) immediately after eating? | □ | □ |
| 8. Do you drink more than 1500CC of plain water every day? | □ | □ |
| 9. Do you like to drink sugary beverages? | □ | □ |
| 10. Do you go for an oral checkup once every six months? | □ | □ |
